# A Cost Effective Block Framing Scheme for Underwater Communication

**DOI:** 10.3390/s111211717

**Published:** 2011-12-16

**Authors:** Soo-Young Shin, Soo-Hyun Park

**Affiliations:** The Graduate School of Business IT, Kookmin University, Jeongneung-ro 77, Seongbuk-gu, Seoul 136-702 Korea; E-Mail: sy-shin@kookmin.ac.kr (S.-Y.S)

**Keywords:** underwater MAC, block framing, ARQ, BA, MA, SMA

## Abstract

In this paper, the Selective Multiple Acknowledgement (SMA) method, based on Multiple Acknowledgement (MA), is proposed to efficiently reduce the amount of data transmission by redesigning the transmission frame structure and taking into consideration underwater transmission characteristics. The method is suited to integrated underwater system models, as the proposed method can handle the same amount of data in a much more compact frame structure without any appreciable loss of reliability. Herein, the performance of the proposed SMA method was analyzed and compared to those of the conventional Automatic Repeat-reQuest (ARQ), Block Acknowledgement (BA), block response, and MA methods. The efficiency of the underwater sensor network, which forms a large cluster and mostly contains uplink data, is expected to be improved by the proposed method.

## 1. Introduction

The development of underwater communication technology and systems using acoustic communication has recently seen significant progress. In particular, researchers are attempting to develop an appropriate modem device for underwater communication, with greatly improved reliability [1]. Presently, most researchers accept that underwater communication of this sort should be possible. One interesting issue associated with this problem involves the improvement of underwater communication Media Access Control (MAC), from the point-to-point type to a network type with many nodes. Considering that the success rate of data transmission of underwater communication is significantly lower than that of above-ground communication, a study to improve communication efficiency by reducing the number of transmissions and the size of the payload required is clearly needed. The principal objective of this research was to develop a technique for the creation of remote underwater communication systems with a centralized and extended network structure, which have Cluster Heads or buoys as gateways, to transmit as much reliable information as possible in one shot. If successful, such a system would constitute a viable solution to the unstable underwater transmission environment, which currently requires a prolonged standby time [2,3].

This paper proposes a framing technique to improve efficiency. The efficient MA transmission technique, which collectively transmits Acknowledgement (Ack) or Negative Acknowledgement (Nack), was used herein. The Cluster Head (CH), which supervises scheduling and data collection, collects data via Uplink transmission within a unit cluster. Ack/Nack, which informs of errors, is included in the Beacon frame. Ack or Nack is selected when broadcasting is conducted in order to attenuate the frame length. In this paper, conventional MA is termed Normal Multiple Acknowledgement (NMA) and the proposed method is referred to as SMA. Ack/Nack only transmits information regarding the existence (or non-existence) of transmission errors. Therefore, there is no need to worry about security issues and simplification is possible by reducing the network complexity associated with frequent data transmission and reception. Additionally, in the case of the MA method, which can significantly reduce the number of transmissions, an efficient method to reduce frame length, which increases according to the number of participating network nodes containing Ack, is proposed.

Section 2 explains related works and Section 3 describes block framing for efficient transmission in an underwater environment. Section 4 describes Beacon and proposed Ack information, an important component of the method proposed herein. Section 5 presents the mathematical model and simulation results. Section 6 presents our conclusions.

## 2. Related Works

### 2.1. Conventional ARQ and BA

**Figure 1.**
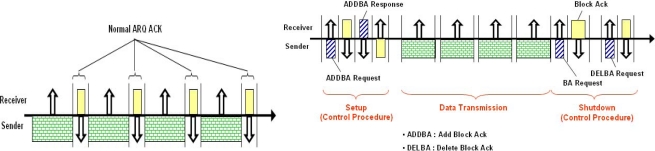
ARQ **(left)** and BA of IEEE 802.11x **(right)**.

The ARQ mechanism was developed for data retransmission in case of a transmission error [4]. The Stop-and-Wait (S&W) ARQ technique is a basic ARQ. With the ARQ, after the sender transmits its data frame, the sender waits an Ack which is a notifying signal of successful reception by the receiver. If there is no Ack message during the pre-defined Ack reception section, or if a Nack is received, the sender will start the procedure of retransmission of the frame (refer to Figure 1). If an Ack is received, the sender will start to transmit the next frame. In this way, the S&W ARQ technique has advantages of simplicity and reliability of data transmission however it has disadvantages of lower effective links in the case of larger packets resulting from a high transmission delay rate as well. In addition, the next data frame cannot be transmitted until receiving an Ack/Nack or a time-out reached. Go-Back-N, Selective Repeat technique, Hybrid ARQ and other techniques have been introduced [5–7].

A study for increasing transmission effectiveness of an ARQ and its practical implementation has been conducted simultaneously. In the IEEE 802.11x standard, the BA technique was proposed to reduce the waste of channels that resulted from Ack transmission. In the proposed technique, MAC Protocol Data Units (MPDUs) are transmitted at an interval of a Short Inter Frame Space (SIFS) period and the BA is transmitted at once after the last data is received [8–10].

There are several big differences between underwater data transmission technology and requirements and the Wireless LAN IEEE 802.11x technology, which makes it possible to transmit data at high speed in wireless radio communication environment. Since the BA technique of IEEE 802.11x standard has been implemented based on the assumption of wideband, the frequency of control frame transmission does not cause difficulty during the procedure of setup and message sequencing for exchanging a BA [8,9]. In an underwater environment, however, the BA technique will cause excessive overhead and the problem of transmission delay underwater cannot be overcome.

The Pervasive Block Ack (PBA) technique has smaller transmission overhead by simplifying the transmission procedures of the BA technique. Channel efficiency and the number of transmissions are significantly improved by the skipping of the transmission of control information notifying the starting and ending of a BA [10]. Further study is necessary to apply BA technology to Under-Water Sensor Networking (UWSN).

### 2.2. The MA Mechanism

**Figure 2.**
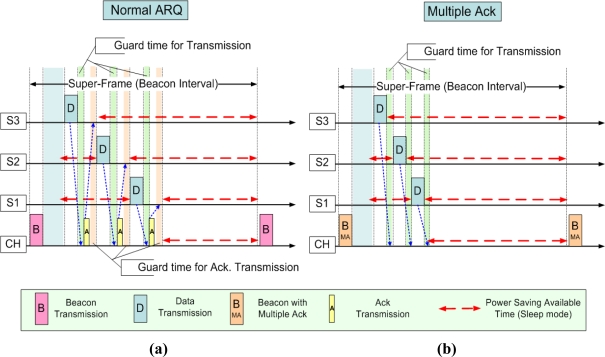
Multiple Access Examples for ARQ **(a)** and MA **(b)**.

The MA mechanism is to simultaneously send an Ack to many receivers. The CH (master, coordinator) of the unit cluster broadcasts a Beacon frame where Ack information of the previously transmitted data is included. Figure 2 shows an example of a normal ARQ and MA technique in a situation of multiple accesses. S1∼S3 are sensor nodes (sender) and CH is a cluster header (receiver). In the case of MA, the frame containing control frames within the super-frame transmitted 4 times (*1 beacon + 3 data*) compared with 7 times of transmission (*1 beacon + 3 data + 3 Acks*) by using ARQ. Red dotted lines in Figure 2 show the margin which can be saved by reduced Ack transmission time and marginally guaranteed Guard time. With the MA technique, the network lifetime can be increased by managing energy consumption. For example, the total number of frame transmissions, the transmission time, and the Guard time can be reduced and energy consumption also can be reduced as much as the reduced duty rate. In addition, the technique can be an efficient method in case of an inferior media environment, such as in underwater [3].

### 2.3. Comparison of Conventional Mechanisms

**Figure 3.**
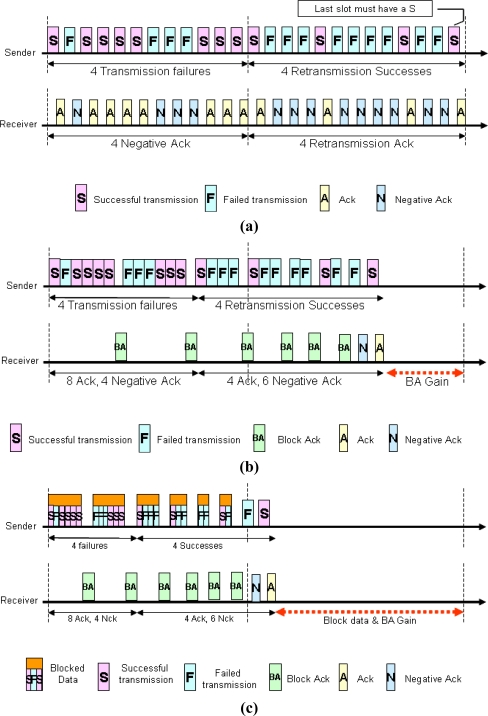
Comparison-1 **(a)** ARQ; **(b)** BA; **(c)** Blocked data & BA.

Figure 3 shows the difference between conventional Ack (ARQ) and blocked Ack. It can be shown that four transmission failures occurred after the first transmission of 12 data frames loaded in 12 time slots. As shown in the figures, the receiver who detected the occurrence of errors transmits Nack and the transmitter restores all the failed data by re-transmission. At this moment, the last time slot must be transmitted successfully and the error rate and the re-transmission rate come to be higher. In case of the link with good quality, the probability of transmission error falls down and the advantage of BA comes to be more remarkable. In case of no transmission errors, the number of transmission of BA is the ratio of the number of A-th transmission 1/(the number of whether the transmission, which is bound as BA, is successful or not) multiplied by (the number of transmission of BA). For example, in case of successful transmission of 12 data in ARQ, which are shown in Figure 3(a), without any transmission failures, 24 of frame transmission, which consists of 12 data and 12 Ack, is required. In case of BA with a 6-block, however, the data being transmitted are reduced to 12 data and 2 BA that the overhead or the number of Ack transmission, is reduced by 1/6 and the overall channel efficiency including data transmission is increased by 117%.

In addition, Figure 3(c) shows that blocked data transmission is more efficient. The number of transmission was reduced by 68% from 25 to 8 and the total number of transmission including Ack was also reduced by 52% from 33 to 16.

**Figure 4.**
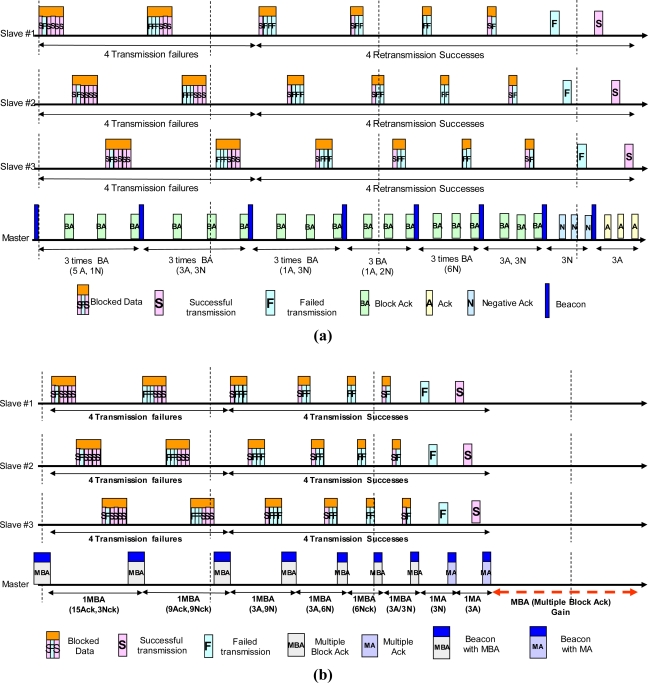
Comparison-2: BA **(a)** *vs.* MBA **(b).**

In case of conducting the Smart Blocking MAC (SBMAC) policy, which is NMA, SMA, Normal Multiple Block Ack (NMBA), Selective Multiple Block Ack (SMBA) policy including Beacon-Control frame, it comes to be possible without additional Ack transmission to notify several nodes of whether or not the transmission succeeded (refer to Figure 4).

Referring to the explanation presented previously, since it is not necessary to transmit in twice for BA, data transmission is performed by twelve times only and Ack is substituted for referring Beacon frame, an improvement of efficiency up to 200% is expected in the number of transmission respect. In addition, the reduction of data size which is resulted from blocking several frames and sharing MAC header is also a good reason for decreasing transmission time. BA, which has been introduced in IEEE 802.11e and IEEE 802.11n, secures ‘BA Gain’ by decreasing transmission time (refer to Figure 4(a)).

“BA’s Gain” on Point-to-Point connection is merely 1/(Number of link connections) of the whole network efficiency. In case of conventional Point-to-Multipoint type sensor network, the efficiency is maximized in proportional to the number of nodes. Figure 4 shows a Master trying to communicate with three Slaves. Communication environment which is supposed in the previous example is applied to each Slave and ‘BA Gain’ is tripled by transmission gains of the three transmitting nodes. Figure 4(b) shows that how much the NMBA or proposed SMBA method is efficient in the same transmission environment previously described.

### 2.4. SCB (Smart Calculation Block) Procedure

**Figure 5.**
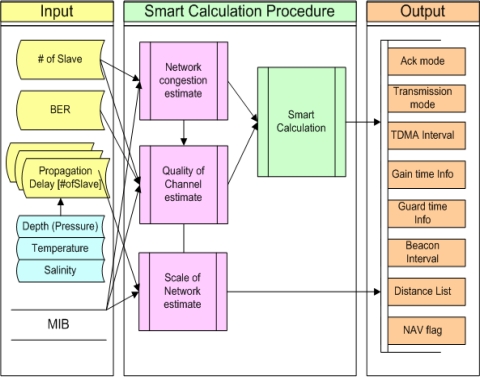
Smart Calculation Procedure of SBMAC.

In this section, the procedure for Smart Calculation of SBMAC is described in detail. By going through SCB, important data which are related to policy of transmission and error restoration, TDMA, congestion control and scheduling, are to be calculated based on many input variables. Figure 5 shows conceptual diagrams of the overall flow of SCB procedures [2].

Firstly, transmission delay time considering the number of Slaves managed by Master, channel’s error rate, water depth, water temperature and salinity is measured and inputted. Management Information Base (MIB), which is a set of variables in MAC, is called out of necessity. Then, in SCB Procedure, Network congestion estimation process, which measures and samples the degree of congestion, and Quality of Channel estimation process, which measures channel quality based on transmission error rate, Scale of Network estimation process, which conducts distance grouping by measuring network scale and calculates TDMA interval, standard value of Gain-time and Guard-time, and other processes are performed [11,12]. Lastly, SCB process is conducted for determination of transmission/Ack mode and error restoration policy.

## 3. Block Framing Scheme

This chapter describes the associated subjects of this research in greater detail. Section 3.1 provides an overview of the basic concepts, and Section 3.2 explains some common components of the frame format. Section 3.3 defines the data transmission frame and frame structure. The example used herein, which was also used as the basis for this research, is an SBMAC, which is specifically designed for an underwater environment.

### 3.1. Basic Concepts and Definitions

The starting point of an SBMAC involves the identification of the sea environment and the calculation of the Guard band, channel quality and bandwidth, and also entails the determination of sea communication policy and important parameters [13,14]. The transmission policy can be categorized into normal data transmission and blocked data transmission, due to the Smart Calculation. In the case of normal data transmission, the receiver can use the NMA or No Acknowledgement (NA) method. In the case of blocked data transmission, the NMBA or NA method can be used. Determination of the Time Division Multiple Access (TDMA) interval, Gain time, Guard time, and Beacon interval are all important concepts; however, this paper focuses specifically on SMA, SMBA frame definition and explanation of the frame structure and its relevance to improved efficiency. Definitions of frame format are listed in Tables 1–8.

Synchronization in an underwater environment is problematic, largely because of the variable delay time. Therefore, it is necessary to implement a blocking algorithm which transmits maximized or optimized packets in a single successful transmission. The received data of the MAC-the inner part of MPDU-will harbor many unit payloads and can acknowledge that the transmission was successful, via the blocking technique.

In the case of the SBMAC underwater sensor network system, Node ID is designed to have up to 127 slaves within a single cluster. In the conventional method, the Type value is set to 0 in the case of Normal data and set to 1 in the case of Block. Ack varies according to the number of Slaves in a cluster. The maximum length of frame section, Len, is 2^7^−1 (127). NMA requires 1 byte per node and NMBA requires 3 bytes per node to transmit Ack information. In the case of NMBA, more than one Ack is required. Although the Bitmap-Ack size is 2 bytes in the present frame format, this size is not fixed. The maximum number of Ack Subsets is 127 (127 × 3 bytes = 381 bytes), as Length is the number of Subsets for Ack. For example, the required lengths of NMA and NMBA would be 10 bytes and 30 bytes, respectively, in a case in which 10 Slaves transmit Acks.

### 3.2. Data Frame

**Figure 6.**
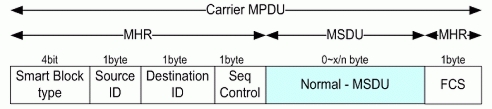
Normal Data Frame format.

**Figure 7.**
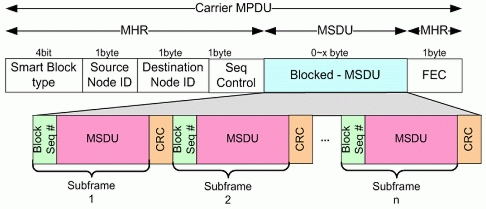
Blocked Data Frame format (Required Block Ack).

Two figures show data frame structures for transmission. Figure 6 is for normal data transmission, and Figure 7 is for blocked data transmission. Beacon frame which has Ack is more important since it has SMA and SMBA mechanism, at which this paper is focusing. That is, conventional NMA and NMBA and new SMA and SMBA use same data frame. The difference of the structure comparing with conventional sensor network system is at the fact that it has minimized Node_ID and Control information and configured compactly to cope with underwater environment.

#### 3.2.1. Normal Data Frame

Figure 6 shows the Normal Data Frame format.

#### 3.2.2. Blocked Data Frame

Figure 7 shows the Blocked Data Frame format.

In the case of the MAC Service Data Unit (MSDU) packing format, which blocks data transmission, the same MAC header can be shared; thus, it is much more efficient than the MPDU blocking technique. Please note that in this method, the transmitter and receiver are the same, as the Source Node ID and Destination Node_ID are the same and all Blocking MSDUs use the Unicast method. A characteristic feature of this underwater sensor network is that periodically collected data are monitored by the user using buoys and satellites. That is, if no special exceptions pertain, the Slave transmits collected data to one Master, and thus the above frame format for efficient system construction is possible.

## 4. Beacon with Proposed Ack frame

### 4.1. Proposed SMA/SMBA

Tables 9–10 show re-defined Ack type and Ack Length for the proposed technique of SMA and SMBA.

In the case of the proposed SMA/SMBA, Ack or Nack is first selected, and then the smaller one can be transmitted. That is, Len will not exceed (number of Slaves)/2 after Type is determined. Len can be expressed up to 63, and Slaves up to 127. The possible size of SMA information is up to 63 bytes. In the case of SMBA, the maximum number of Ack-subsets is 63 (3 × 63 bytes = 189 bytes). For example, if there are three Nacks among 10 Slaves, three bytes for SMA information and nine bytes for SMBA are required.

In a case wherein all 63 Slaves should receive BA replies, however, the probability of transmission error can increase, owing to the increased variable frame length. Since an optimized length of frame has already been used in computation, if the length of MBAs exceeds the pre-determined frame length, Network Allocation Vector (NAV) flag 1110 provides a method for the splitting and transmission of Ack information continuously [5].

### 4.2. Beacon Frame with Variable Acks

The proposed SMA and SMBA mechanism is a technique of optimizing the frame size of Beacon which is transmitted in MA technique. Its difference from conventional NMA and NMBA can be seen from the figure below and explanations.

**Figure 8.**
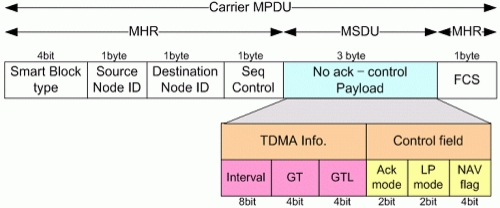
Beacon Frame with NA.

**Figure 9.**
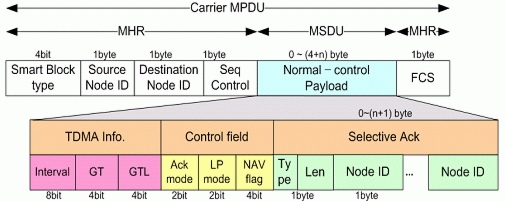
Beacon Frame with SMA.

**Figure 10.**
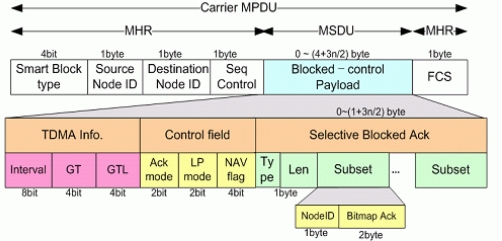
Beacon Frame with SMBAs.

The proposed system can send Ack information or SMA in case of normal transmission or SMBA in case of blocked transmission according to various strategic variables. Figures 8–10 show Beacon format having Ack. Figure 8 is for NA without Ack about transmitted data. And Figure 9 is for SMA in normal case. Figure 10 is for SMBA in case of blocked data. Receivers of Ack informations will analyze Ack mode information of Control field, sense the types of Ack and conduct appropriate procedure.

#### 4.2.1. Beacon Frame with NA

Figure 8 shows a format of a Beacon frame with NA information. In case of NA, which transmit without Ack information, NMA, SMA, NMBA and SMBA will have the same size since they all have not Ack field. Ack mode will set to 00 in case of normal data and 01 in case of blocked data.

#### 4.2.2. Beacon Frame with SMA

Figure 9 shows a format of a Beacon frame with SMA information about Normal data. In case of SMA, which transmit data with Ack information of Normal data, the size of SMA Ack field will be 1/2 compared with NMA. Ack/Nack information will be set in Type field.

#### 4.2.3. Beacon Frame with SMBA

Figure 10 shows the A frame of the Beacon frame with SMBA information. SMBA has information for transmission of Bitmap Blocked Ack about Blocked data to multiple node simultaneously. The number of Subset will be changed according to their Type.

## 5. Computational Model and Numerical Results

### 5.1. Definitions

In this section, the analytical formula is defined to form the theory of the proposed SMA definitions (refer to Table 11). The terms are as follows:

### 5.2. Numerical Computation Model

Channel usability can be expressed as *R/C*—Frame transmission rate over the total bandwidth. The efficiency of the channel being used means the rate of data length over the total transmitted frame. This can be expressed by $\frac{L_{\mathrm{payload}}}{L_{\mathrm{total}}}=\frac{L_{\mathrm{total}}-L_{\mathrm{control}}}{L_{\mathrm{total}}}$. The length of the total transmitted frame is the payload length plus the control information length. The control information length is expressed in Equations (1) and (2):

(1) $$L_{\mathrm{total}}=L_{\mathrm{payload}}+L_{\mathrm{control}}$$

(2) $$L_{\mathrm{control}}=(L_{\mathrm{data}}-L_{\mathrm{payload}})+L_{\mathrm{ack}}+\mathrm{BEACON}$$

Equations (3) and (4) show the component fields in the conventional Ack and data transmission Frame. Four variants of Ack length are expressed in Equation (3). Equations (4–7) show the ARQ, BA, NMA, and proposed SMA method, respectively. The size of the address and control field are the same for fair comparison. The number of frames included in one block is assumed to be 5 for numerical calculation. The number of slaves in the network is also 5 (for example):

(3) $$\mathrm{Len}({\mathrm{ACK}}_{\mathrm{SMA}})<\mathrm{Len}({\mathrm{ACK}}_{\mathrm{NMA}})<\mathrm{Len}({\mathrm{ACK}}_{\mathrm{ARQ}})<\mathrm{Len}({\mathrm{ACK}}_{\mathrm{BA}})$$

(4) $$\begin{array}{c} {\mathrm{ACK}}_{\mathrm{ARQ}}=\mathrm{Frame Control}+\mathrm{Duration ID}+\mathrm{Dstination ID}+\mathrm{CRC}\\ \text{and multiple}\;\#\;\text{of Slaves},\;\#\;\text{of tx in one block}/\text{ACK}\_\text{frame}\\ \mathrm{Example}:{\mathrm{ACK}}_{\mathrm{ARQ}}=1+1+1+1=4\;\mathrm{bytes}=32\;\mathrm{bits},\;32\times 5\times 5=800\;\mathrm{bits} \end{array}$$

(5) $$\begin{array}{c} {\mathrm{ACK}}_{\mathrm{BA}}=\mathrm{Frame Control}+\mathrm{Duration ID}+\mathrm{Destination ID}+\\ (\mathrm{BA Control}+\mathrm{Block Ack Sequence Control}+\mathrm{Block Ack Bitmap})+\mathrm{CRC}\\ \text{and Multiple}\;\#\;\text{of Slaves}/\text{Block}\_\text{ack}\_\text{frame}\\ \mathrm{Example}:{\mathrm{ACK}}_{\mathrm{BA}}=1+1+1+(1+1+1)+2=8\;\mathrm{bytes}=64\;\mathrm{bits},64\times 5=320\;bits \end{array}$$

(6) $$\begin{array}{c} {\mathrm{ACK}}_{\mathrm{NMA}}=\mathrm{Normal Ack field}(\mathrm{Type}+\mathrm{Len}+(\mathrm{Corresponding ID}\times \#\;\mathrm{of Slaves}))\\ \text{and multiple}\;\#\;\text{of tx in one block}/\text{NMA}\_\text{ack}\_\text{fields}\\ \mathrm{Example}:{\mathrm{ACK}}_{\mathrm{NMA}}=1+(1\times 5)=6\;\mathrm{byte}=\mathrm{48}\;\mathrm{bits} \end{array}$$

(7) $$\begin{array}{c} {\mathrm{ACK}}_{\mathrm{SMA}}=\mathrm{Selective Ack field}\;(\mathrm{Type}+\mathrm{Len}+(\mathrm{Corresponding ID}\times \mathrm{int}(\#\;\mathrm{of Slavce}/2))\\ \text{and multiple}\;\#\;\text{of tx}\;\text{in one block}/\text{SMA}\_\text{ack}\_\text{fields}\\ \mathrm{Example}:{\mathrm{ACK}}_{\mathrm{SMA}}=1+2=3\;\mathrm{byte}=24\;\mathrm{bits} \end{array}$$

The data frame lengths of ARQ, BA, NMA and SMA are the same. This means that channel efficiency is derived from the difference of the Ack methods and control frame length. Thus, data.ARQ, data.BA and data.MA denote the Data Frames for ARQ, BA, NMA and SMA, respectively. For example, the transmitted data length, Payload, is established at eight bytes:

(8) $$L_{\mathrm{data}.\mathrm{SMA}}=L_{\mathrm{data}.\mathrm{NMA}}=L_{\mathrm{data}.\mathrm{BA}}=L_{\mathrm{data}.\mathrm{ARQ}}$$

(9) $$\begin{array}{c} \mathrm{data}.\mathrm{ARQ}=\mathrm{data}.\mathrm{BA}=\mathrm{Frame Control}+\mathrm{Duration ID}+\mathrm{Source ID}+\\ \mathrm{Destination ID}+\mathrm{Sequence Control}+\mathrm{Payload}+\mathrm{CRC}\\ \mathrm{Example}:\mathrm{data}.\mathrm{ARQ}=\mathrm{data}.\mathrm{BA}=1+1+1+1+1+8+1=14\;\mathrm{bytes}=\mathrm{112}\;\mathrm{bits} \end{array}$$

(10) $$\begin{array}{c} \mathrm{Data}.\mathrm{SMA}=\mathrm{Data}.\mathrm{NMA}=\mathrm{SmartBlock}+\mathrm{SourceID}+\\ \mathrm{DestinationID}+\mathrm{Sequence Control}+\mathrm{Payload}+\mathrm{CRC}\\ \mathrm{Example}:\mathrm{data}.\mathrm{SMA}=\mathrm{data}.\mathrm{NMA}=4\mathrm{bits}+1+1+1+8+1=\mathrm{12}.5\;\mathrm{bytes}=\mathrm{100}\;\mathrm{bits} \end{array}$$

The number of transmissions of the Ack Frame and Control Frame and the total length of messages are explained. Equations (11) and (12) are for the ARQ method, Equations (13) and (14) are for the BA method, Equations (15) and (16) are for Multiple Ack, and Equations (17) and (18) are for the Selective Multiple Ack method. The number of data transmissions is 100:

(11) $$\begin{array}{c} N_{\mathrm{ack}.\mathrm{ARQ}}=N_{\mathrm{data}}\\ \mathrm{Example}:N_{\mathrm{ack}.\mathrm{ARQ}}=N_{\mathrm{data}}=100 \end{array}$$

(12) $$\begin{array}{c} \sum L_{\mathrm{ack}.\mathrm{ARQ}}=\mathrm{Len}({\mathrm{ACK}}_{\mathrm{ARQ}})\cdot N_{\mathrm{ack}.\mathrm{ARQ}}\\ \mathrm{Example}:\Sigma L_{\mathrm{ack}.\mathrm{ARQ}}=5\times \mathrm{100}=\mathrm{500} \end{array}$$

*N_data_* is the number of data transmissions and *N_data_/B* is used to calculate the number of transmissions of BA. The number is converted into an integer value by *int*(). In Equation (13), the number of transmissions is multiplied by 3 due to the two additional frames required for the start and end of *BA* (*=SET_BA_*):

(13) $$\begin{array}{c} N_{\mathrm{ack}\mathrm{.BA}}=3\cdot \mathrm{int}(\frac{N_{\mathrm{data}}}{B})\\ \mathrm{Example}:N_{\mathrm{ack}.\mathrm{BA}}=3*\mathrm{int}(100/5)=60 \end{array}$$

(14) $$\sum L_{\mathrm{ack}.\mathrm{BA}}=\mathrm{Len}({\mathrm{ACK}}_{\mathrm{BA}})\cdot \mathrm{int}\left(\frac{N_{\mathrm{data}}}{B}\right)+2\cdot \mathrm{Len}({\mathrm{SET}}_{\mathrm{BA}})\cdot \mathrm{int}(\frac{N_{\mathrm{data}}}{B})$$

MA does not need to transmit additional control frames, such as *SET_BA_* for Ack. This efficiency improvement is the consequence of the minimization of information inside Ack and Data. There is no transmission number for Ack in the cases of NMA and SMA, since all *ACK* information is transmitted with *BEACON*. Additionally, the sum of the *ACK* length is less than that in the other three methods. Equations (15–17) are for NMA and SMA, respectively:

(15) $$N_{\mathrm{ack}.\mathrm{NMA}}=0$$

(16) $$\sum L_{\mathrm{ack}.\mathrm{NMA}}=\mathrm{Len}(\mathrm{BEACON}(\mathrm{Normal}.\mathrm{Ack}.\mathrm{Field}))$$

(17) $$\sum L_{\mathrm{ack}.\mathrm{NMA}}=\mathrm{Len}(\mathrm{BEACON}(\mathrm{Selective}.\mathrm{Ack}.\mathrm{Field}))$$

### 5.3. Numerical Results

**Figure 11.**
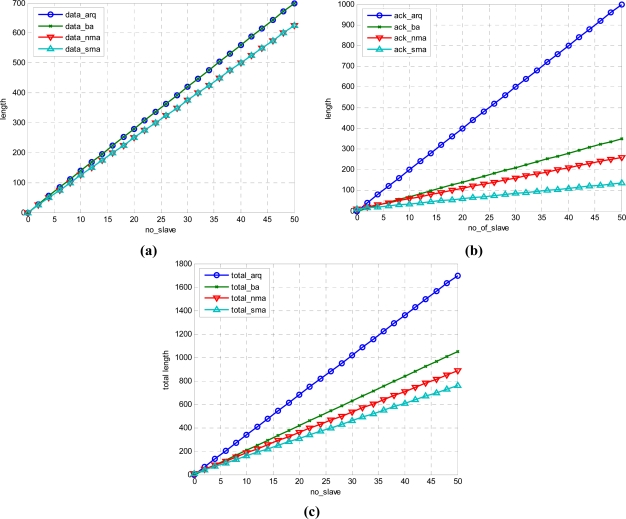
Frame Length **(a)** data length **(b)** ack length **(c)** total length.

Figure 11 shows the numerical result of the above equations.

Figure 11 shows the data frame, frame length of Ack and control frame, and total length. The data show a relatively small number in nma and sma. arq and ba have the same length. However, in the case of ack, the proposed sma has the smallest length for the same information. In this paper, a technique for significant reduction of frame size was proposed. Although nma and sma in Figure 11(a) is same, the length of Ack is reduced significantly as shown in Figure 11(b) so that the whole length is reduced as shown in Figure 11(c).

**Figure 12.**
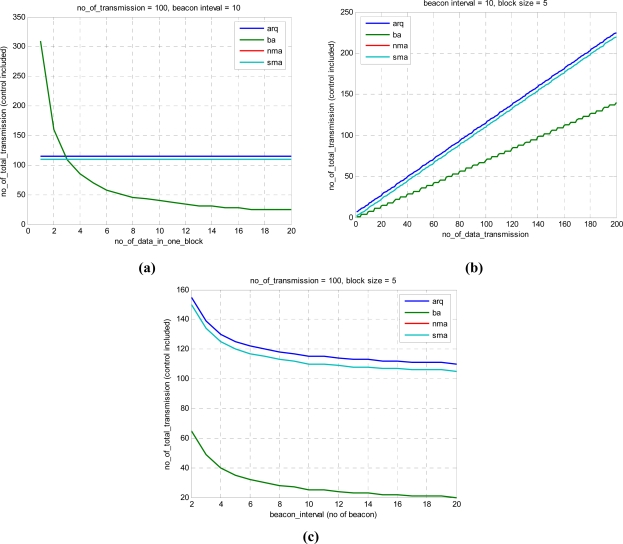
Number of total transmission: **(a)** variable block **(b)** variable data tx **(c)** variable beacon interval.

Figure 12 shows the variation of transmission number for many variables. The reduction in transmission number is meaningful in underwater communication, in which minimizing the number of transmissions is important. Figure 12(a) is for various numbers of data in a block. Figure 12(b) is dependent on the frequency of data transmission. Figure 12(c) is for the transmission period and transmission number. The proposed sma evidences the best performance.

**Figure 13.**
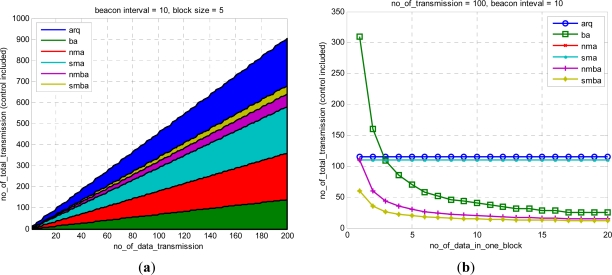

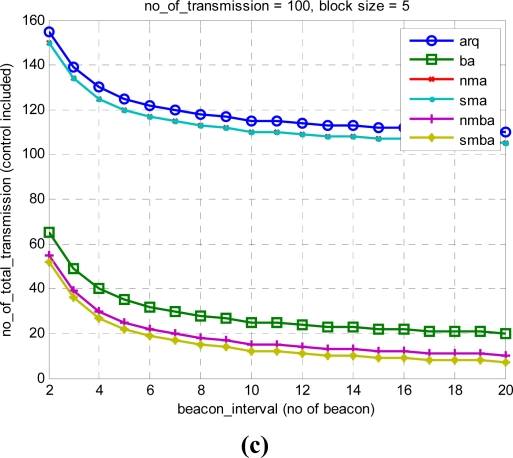
Number of Total Transmissions **(a)** variable tx interval **(b)** variable block size **(c)** variable beacon interval.

Figure 13 shows the number of total transmissions using 6 methods. nmba and smba denote the NMBA method and SMBA method, respectively. The proposed method evidences performance superior to that achievable with the conventional BA method. The number of transmission is an important factor especially in underwater environment. The proposed smba showed the smallest number of transmission and as data transmission interval, Beacon interval and the size of data in a block increases the performance is improved more.

## 6. Conclusions

This paper introduces a framing technique to reduce transmission data using the new MA concept. The efficiency of the conventional Ack method can be improved and overhead can be significantly reduced via the proposed method. In the case of MA, there is no transmission specifically for Ack. Ack information of all nodes within unit transmission range is included in a periodic network Beacon. The performance of ARQ, BA, NMA, SMA, NMBA and SMBA was also assessed in this study.

Optimization of frame size and the number of transmission is an important factor for performance, especially in an underwater environment. As simulation results shows, the proposed SMA and SBMA present the smallest frame size and the lowest number of transmission with various variables. As the number of transmission targets increases, the performance of the proposed technique shows better performance. This is resulted from the optimized frame size producing reduced error rate and efficient transmission.

In future research, the Ack information reduction technique will be developed further, and more detailed performance analyses between methods will be conducted. In addition, an underwater test environment will be constructed for field test and performance verification.

## Figures and Tables

**Table 1.** Smart Block type.

**Smart Block type**	**Definition**

0000	Beacon frame with NMBA (Master)
0001	Beacon frame with NMA (Master)
0010	Beacon frame with NA (Master)
0011	Data frame with Blocked MSDU (Master/Slave)
0100	Normal Data frame (Master/Slave)
0101	Init. Request frame with sync. (Master)
0110	Init. Response frame with mac address (Slave)
0111	Init. Confirm frame with unique ID mapping list (Master)

**Table 2.** Node ID.

**ID**		**Definition**
Cluster ID	Node ID	
00∼11	000000	Master
	000001∼111111	Slave (2^6^−1)

**Table 3.** TDMA Info.

TDMA Info.	Interval	0x00∼0xff	Transmission Interval on TDMA (time slots)
	GT	0x0∼0xf	Gain time of Propagation Delay (time slots)
	GTL	0x0∼0xf	Guard time for Listening (time slots)

**Table 4.** Ack mode (NMA/NMBA).

Ack mode (2 bits)	00	Normal Data transmission & NA
	01	Blocked Data transmission & NA
	10	Normal Data transmission & NMA
	11	Blocked Data transmission & NMBA

**Table 5.** LP mode.

LP Mode (2 bits)	00	Not use Low Power mode
	01	Use Low Power mode
	10	Reserved
	11	Reserved

**Table 6.** NAV flag.

NAV Flag (4bits)	Non transmission mode	0000	1 Level Deadlock state for error control
		0001	2 Level Deadlock state for error control
		0010	3 Level Deadlock state for error control
		0011	4 Level Deadlock state for error control
		0100	5 Level Deadlock state for error control
		0101	6 Level Deadlock state for error control
		0110	7 Level Deadlock state for error control
		0111	8 Level Deadlock state for error control
	Transmission mode	1000	Normal transmission state
		1001	Reconfiguration of network
		1010	Join of new Slave
		1011	Master --> Slave data transmission
		1100	Master --> Relay node data transmission
		1101	Master --> GW data transmission
		1110	Another Beacon transmission
		1111	Reserved

**Table 7.** Ack type.

Type	0	NMA (Node ID) Transmission
	1	NMBA (Node ID, BitmapAck) Transmission

**Table 8.** Ack Length.

Len	0∼(2^7^−1)	NMA/NMBA : # of (Block) Ack - NMA : Node ID 1byte/Ack - NMBA : Node ID, BitmapAck 3bytes

**Table 9.** Ack type (SMA/SMBA).

Type	00	SMA (Node ID) Ack Transmission
	01	SMA (Node ID) Nack Transmission
	10	SMBA (Node ID, BitmapAck) Transmission
	11	SMBA (Node ID, BitmapNack) Transmission

**Table 10.** Ack Length (SMA/SMBA).

Len	0∼(2^6^−1)	SMA/SMBA: # of (Block) Ack - SMA : less then (Node ID 1byte/Ack or Nack)/2 - SMBA :less then (Node ID, Bitmap-ack 3bytes)/2

**Table 11.** Definitions.

**Notation**	**Definition**

*C*	Network Bandwidth
*R*	Data Rate
*B*	Number of Blocking Acks
*data*	Data Frame with control information
*SET*	BA control Frame
*BEACON*	Master driven periodic Broadcasting Frame
*ACK*	ACK Frame
*L_total_*	Length of total frame (Data frame + Ack frame)
*L_data_*	Length of *data*
*L_payload_*	Length of MSDU(Payload)
*L_control_*	Length of control
*L_ack_*	Length of *ACK*
*ΣCK_ack_*	Total length of *ACK* on link
*N_total_*	Number of total T_x_
*N_data_*	Number of *data* T_x_
*N_ack_*	Number of *ACK* T_x_
*N_control_*	Number of control T_x_ (BA association + disassociation)
*Len()*	Function of frame length
*int()*	Function of integer
